# Digital Expression Profiling Identifies *RUNX2*, *CDC5L*, *MDM2*, *RECQL4*, and *CDK4* as Potential Predictive Biomarkers for Neo-Adjuvant Chemotherapy Response in Paediatric Osteosarcoma

**DOI:** 10.1371/journal.pone.0095843

**Published:** 2014-05-16

**Authors:** Jeffrey W. Martin, Susan Chilton-MacNeill, Madhuri Koti, Andre J. van Wijnen, Jeremy A. Squire, Maria Zielenska

**Affiliations:** 1 Department of Paediatric Laboratory Medicine, Hospital for Sick Children, Toronto, Ontario, Canada; 2 Departments of Orthopedic Surgery and Biochemistry and Molecular Biology, Mayo Clinic, Rochester, Minnesota, United States of America; 3 Department of Pathology and Molecular Medicine, Queen’s University, Kingston, Ontario, Canada; 4 Department of Biomedical and Molecular Sciences, Queen’s University, Kingston, Ontario, Canada; 5 Departments of Genetics and Pathology, Faculdade de Medicina de Ribeirão Preto - USP, Ribeirão Preto, São Paulo, Brazil; Johns Hopkins University, United States of America

## Abstract

Osteosarcoma is the most common malignancy of bone, and occurs most frequently in children and adolescents. Currently, the most reliable technique for determining a patients’ prognosis is measurement of histopathologic tumor necrosis following pre-operative neo-adjuvant chemotherapy. Unfavourable prognosis is indicated by less than 90% estimated necrosis of the tumor. Neither genetic testing nor molecular biomarkers for diagnosis and prognosis have been described for osteosarcomas. We used the novel nanoString mRNA digital expression analysis system to analyse gene expression in 32 patients with sporadic paediatric osteosarcoma. This system used specific molecular barcodes to quantify expression of a set of 17 genes associated with osteosarcoma tumorigenesis. Five genes, from this panel, which encoded the bone differentiation regulator *RUNX2*, the cell cycle regulator *CDC5L*, the *TP53* transcriptional inactivator *MDM2*, the DNA helicase *RECQL4*, and the cyclin-dependent kinase gene *CDK4*, were differentially expressed in tumors that responded poorly to neo-adjuvant chemotherapy. Analysis of the signalling relationships of these genes, as well as other expression markers of osteosarcoma, indicated that gene networks linked to RB1, TP53, PI3K, PTEN/Akt, myc and RECQL4 are associated with osteosarcoma. The discovery of these networks provides a basis for further experimental studies of role of the five genes (*RUNX2*, *CDC5L*, *MDM2*, *RECQL4*, and *CDK4*) in differential response to chemotherapy.

## Introduction

Osteosarcoma is the most common primary malignant bone tumor arising from bone in children and adolescents. The tumor is very rare with an incidence approaching 5 per million per year [1]. Tumors arise from mesenchymal cells predominantly in the metaphyses of the distal femur, proximal tibia, and proximal humerus adjacent to epiphyseal growth plates [2]. In rare cases, osteosarcomas affect the axial skeleton and other non-long bones [1]. Chemotherapy followed by surgical resection is the standard treatment for high-grade osteosarcoma, and the current drug regimen is a combination of high-dose methotrexate, doxorubicin, cisplatin, and ifosfamide.

Histopathologic examination to estimate tumor necrosis following neo-adjuvant pre-operative chemotherapy is currently one of the most reliable tools for response evaluation and prognostication. Unfavorable response, corresponding to a bad prognosis, is indicated by less than 90% estimated necrosis of the tumor following neo-adjuvant chemotherapy [1]. Unlike other paediatric cancers, there are few consistent genomic translocations, amplifications, or deletions in osteosarcoma that are useful for clinical diagnosis/treatment [3]. Similarly, a large number of gene products have potential for driving oncogenesis or disease progression in osteosarcoma [4]. This complex biology of osteosarcoma has limited the identification of reliable molecular biomarkers for tumor classification or therapeutic targeting.

Definitive diagnosis of osteosarcoma requires the presence of immature osseous matrix around neoplastic cells, which are often osteoblast-like. The predominant epithelial mesenchymal lineages define the tumor subtype based on the main form of extracellular matrix observed: osteoblastic osteosarcoma (osteoid matrix), chondroblastic osteosarcoma (cartilaginous tissue), or fibroblastic osteosarcoma (fibrous tissue). The histological subtype may define specific molecular pathways involved in osteosarcoma development and progression [4]. The high level of genetic and cytogenetic heterogeneity of this tumor, both between patients and within the tumors themselves [5], necessitates specific and personalised treatment approaches to improve outcomes.

Paediatric osteosarcoma represents a challenge to cancer treatment teams and research groups alike, a situation that is contrary to other sarcoma types for which expression signatures exist [6]. In fact, a large proportion of other sarcomas are characterised by a single dominant-acting fusion protein encoded by a disease-specific chromosome translocation, while osteosarcoma cells possess cytogenetically complex karyotypes with no such consistent translocations [7]. Scott et al. used a comparative biology approach to discover molecular subtypes of human osteosarcoma after studying profiles of canine osteosarcoma [8]. RT-PCR- and gene expression microarray-based studies of paediatric osteosarcoma have previously been used to investigate disease-specific expression patterns and signatures [9]–[12].

Our previous work revealed significant changes in a number of genes involved in tumor suppressive pathways, cell cycle control, and oncogenic mechanisms [10]. In the present study, candidate genes were selected based on our previous work, as well as on the published reports on gene products with potential for involvement in osteosarcoma development. NanoString nCounter Technology, which has been used previously to classify other tumors [13], was used to determine expression levels of RNA from our cohort of 32 osteosarcoma patients. The nanoString Gene Expression Assay is a high-sensitivity, multiplexed method utilizing specific molecular bar codes for the detection of mRNAs that eliminates any enzymatic reactions [14]. An analysis of the interaction of the most prominent biomarkers in this study with some of the other established oncogenic drivers in osteosarcoma was performed to determine which regulatory networks may underlie the varying responses to neo-adjuvant chemotherapy in this cohort.

## Methods

### Ethics Statement

The 32 patient cohort sample used in this study were obtained according to the guidelines and approval of the Sick Kids Hospital Research Ethics Board. Informed written consent to participate in this study was obtained from the patients, or in the case of young children, their next of kin, caretakers, or guardians on their behalf.

### Paediatric Osteosarcoma and Normal Human Osteoblast Samples

Forty sporadic paediatric osteosarcoma tumor samples derived from a cohort of 32 patients were taken from pre-chemotherapeutic biopsies or from surgical resection specimens. All specimens were flash-frozen following the biopsy procedure or surgical resection. All surgical resection specimens had been exposed to the same standard regimen of neo-adjuvant chemotherapy, including methotrexate, doxorubicin, cisplatin, and ifosfamide. For eight patients, the initial diagnostic biopsy samples, which were naïve to neo-adjuvant chemotherapy, and a matched post-chemotherapy resection sample, collected on average 3.8 months after the first sample, were analysed. These eight pairs of RNA samples were used in a subset analysis to calculate the relationships of expression changes that occurred during the neo-adjuvant chemotherapy. Chemotherapy response of <90% necrosis was used to identify those tumors with a bad response following neo-adjuvant chemotherapy, and these values, along with other patient characteristics, are summarised in Table 1. Normal human osteoblasts isolated from surgical bone resections from five healthy individuals were obtained from PromoCell (Heidelberg, Germany) and combined and run as a single pooled sample. Total RNA was extracted from the tissues using the TRIzol Reagent method according to the manufacturer’s protocol (Invitrogen) and quantified using the Bioanalyzer (Agilent Technologies). Total RNA from normal human osteoblasts and osteosarcoma cell lines was retrieved as described previously [11]. All aliquots were diluted to a final concentration of 20 ng/µL.

**Table 1:** Clinical characteristics of the 32 patients.

Parameter		Number	Percentage
Histopathologic subtype†	Osteoblastic	22	69
	Chondroblastic	4	13
	Fibroblastic	3	9
	Mixed††	3	9
Gender	Female	17	53
	Male	15	47
Type of sample	Resection	21	66
	Biopsy	11	34
Percent necrosis post-chemotherapy	<90%	19	59
	>90%	13	41

† All of the tumors were high-grade.

†† The pathology report described pleomorphic, undifferentiated cells in a tumor comprising cells representing various subtypes.

### nanoString nCounter Assay

The nanoString nCounter gene expression system (nanoString Technologies) was used for expression profiling of the osteosarcomas and normal human osteoblasts. Details of the system are described elsewhere [14]. Briefly, unique multiplexed probes were made with two sequence-specific probes per target mRNA. Two probes were constructed complementary to a 100-base target region. The capture probe comprised a target-specific oligonucleotide coupled to a short sequence linked to biotin. The reporter probe consisted of a second target-specific oligonucleotide linked to a unique chain of dye-labelled RNA segments for detection by the system. Our nCounter code set consisted of 21 probes, including 18 test probes derived from 17 distinct genes (RUNX2 comprised P1 and P2 transcripts) and three control genes (Table S1). Each sample was hybridised in duplicate or triplicate using 100 ng total RNA per reaction, in addition to the capture and reporter probes, as previously described [14].

### Development of Candidate Gene List and nanoString Code Set Design

We selected 17 candidate genes for this study based on published reports describing gene products with the potential for involvement in osteosarcoma development, and based on our own findings (Table 2). The literature we considered, included gene copy number and gene expression microarray experiments, in addition to functional assays of genes, in models of osteosarcoma. In addition we performed pathway analysis using Ingenuity Pathway Analysis (IPA) to delineate overrepresented gene networks in the candidate genes associated with osteosarcoma oncogenesis. IPA employs the Fisher’s exact test to determine the relationship between the input dataset and canonical pathways with associated biofunctions (Ingenuity Pathway Analysis system; http://www.ingenuity.com/). Statistically significant overexpression in osteosarcoma tumors relative to normal osteoblasts has been detected previously for *RECQL4*, *RUNX2*, and *SPP1* [10], [15], as is the case for amplification-related overexpression of *CDC5L* and *RUNX2* osteosarcoma specimens [9]. We included probes for each of the two *RUNX2* transcript isoforms in the codeset. *RUNX2_P1* captures expression of the normal osteoblast-specific version of RUNX2 [16], whereas *RUNX2_P2* captures expression of RUNX2 during earlier stages of development. The latter version is also highly expressed in tumors, such as osteosarcomas [10]. Unless otherwise specified as RUNX2 (P1), all of the reported expression of *RUNX2* refers to the P2 transcript. Over-expression of the protein products of *FOS*, *MYC*, *MDM2*, *CDK4*, *SPARC*, and *BCL2L1* have also been associated with osteosarcoma and have well-described tumorigenic potential [17]–[21]. On the other hand, a high frequency of genetic inactivation and copy number loss in osteosarcoma has been documented for *TP53*, *CDKN2A*, *RB1*, *PTEN*, and *WWOX* [22]–[27]. We included *CDKN1A* and *CDKN1C* in our analysis because of their roles in *TP53* and *RB1*-mediated control of cellular proliferation [28]. We selected *HMBS*, *MT-ATP6*, and *MT-CO1* as housekeeping controls for our experiments because of validation in previous experiments [29]–[31].

**Table 2:** List of experimental genes and control genes assayed.

*MYC*	*RB1*	*TP53*
*FOS*	*CDKN1C*	*p16INK4*
*CDKN1A*	*SPARC*	*RECQL4*
*RUNX2 (P1)*	*SPP1*	*PTEN*
*RUNX2 (P2)*	*BCL-xL*	*HMBS* (control)
*CDC5L*	*MDM2*	*MT-ATP6* (control)
*WWOX*	*CDK4*	*MT-CO1* (control)

Twenty one probes for 17 genes and three controls were assayed for expression in the nanoString code set. Probes for each of the two main promoter regions of *RUNX2* were constructed.

### Statistical Analysis

All data analysis was performed using the nSolver Analysis Software (nanoString Technologies). Briefly, counts are normalised for all target RNAs in all samples based on the positive control probes to account for differences in hybridisation efficiency and post-hybridisation processing, including purification and immobilisation of complexes. The software calculates the geometric mean of each of the controls for each sample to estimate the overall assay efficiency. Subsequently, mRNA content normalisation was performed using the housekeeping “calibration” genes, *MT-ATP6*, *MT-CO1*, and *HMBS*. Values <0 were blanketed and considered equal to 1 to facilitate downstream statistical analyses. All expression data can be accessed through GEO (Accession Number GSE45275). Following normalization (Table S2A and S2B) and removal of the samples with poor quality control data, the unpaired two-tailed Student’s t-test (Table S3) was applied to derive genes that had significant (p<0.05) differential expression in the two groups (25 with poor prognosis and 15 with good prognosis).

## Results

### Correlations of Expression Change with Tumor Response to Chemotherapy

**Figure 1:**
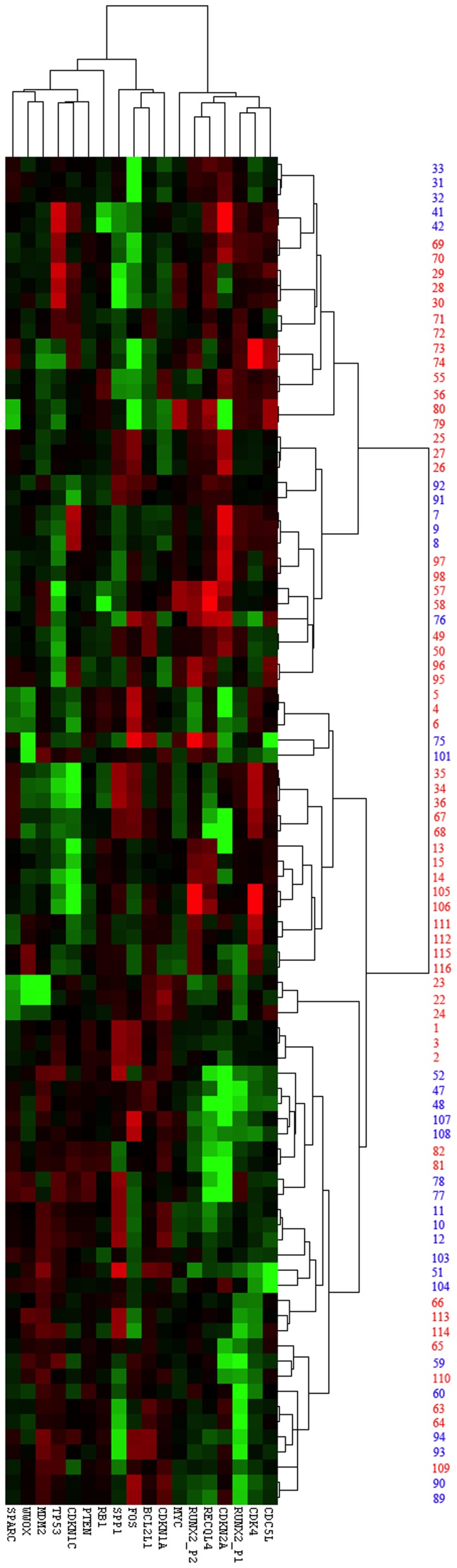
Cluster map constructed using Cluster 3.0, showing the differential expression of the 17 gene set in osteosarcoma biopsy and resection cases in the cohort. Patients with >90% tumor necrosis in response to chemotherapy (good response) are shown in blue and those with <90% (poor response) are shown in red. Sample numbers 1–36 were analyzed in triplicates whereas samples 41–116 were analysed in duplicates. Detailed data manipulation for the cohort is presented in Table S2B.

**Figure 2:**
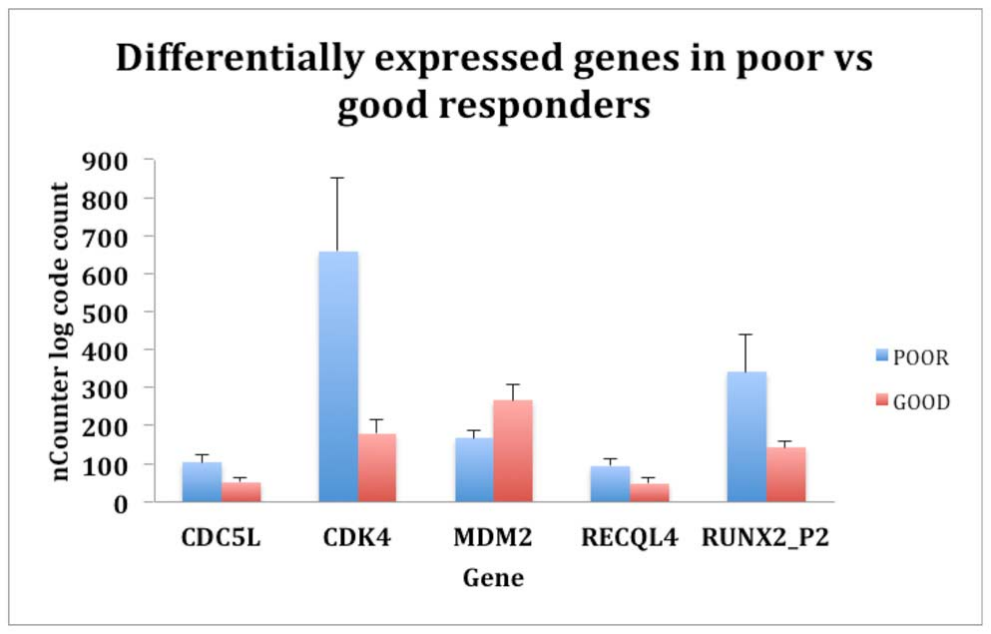
Mean expression of the most significantly discriminating five genes in the osteosarcoma cohort when poor response (<90% tumor necrosis in response to chemotherapy) was compared to good response (>90% tumor necrosis). Unpaired Student’s t-test was applied to derive the genes that were differentially expressed in the two groups.

An unsupervised clustering was performed using Cluster 3.0 and Java tree view to determine the aggregation of the 40 RNA samples (in duplicates or triplicates) from the cohort in comparison to normal human osteoblasts and three osteosarcoma cell lines (Figure S1). An unsupervised analysis was repeated using only tumor samples from the cohort. The cluster map (Figure 1) shows the differential expression of the 17-gene probe code set from our nanoString panel, comparing the good to the poor responders. For the eight paired patient samples, only the initial pre-chemotherapy biopsies were used to examine expression levels of the gene set. Tumors with <90% necrosis in response to neo-adjuvant chemotherapy possessed significantly higher expression levels of: *RUNX2*, *CDC5L*, *CDK4*, and *RECQL4;* and significantly reduced levels of *MDM2* (all p≤0.05) (Figure 2).

### Gene Expression Changes Relative to Normal Human Osteoblasts

Using the duplicate measurements for each experiment, expression changes for 32 patient samples were normalised, averaged, and then ratios relative to normal human osteoblasts were calculated. Statistically significant up-regulated expression in tumors relative to human osteoblasts was detected for *CDKN1C*, *FOS*, *MYC*, *RECQL4*, *RUNX2*, *SPARC*, *SPP1*, and *WWOX.* Down-regulation was observed for *CDKN1A* and *TP53* (Figure S2). Mean expression change and direction relative to normal osteoblasts match our previously published mRNA qRT-PCR analyses for *CDKN1A*, *MYC*, *RUNX2*, and *SPP1* [10].

### Gene Expression Levels Following Exposure to Neo-adjuvant Chemotherapy

For the eight patients with biopsies naïve to chemotherapy and matched resections, it was possible to perform a subset analysis to determine expression changes in resected samples relative to biopsies. mRNA expression was elevated for *BCL2L1* (p<0.05), *CDKN1A* (p<0.05), *MDM2* (p<0.05), *PTEN* (p<0.05), and *WWOX* (p<0.05). No significant differences were seen in the expression levels of *RUNX2*, *CDC5L*, *CDK4*, or *RECQL4*, between biopsy and resection specimens. Of the five genes for which differential expression in tumors was associated with a poor response to chemotherapy, only *MDM2* levels changed after exposure to neo-adjuvant chemotherapy in this subset of eight patients.

### Pathway-dependent Expression

**Figure 3:**
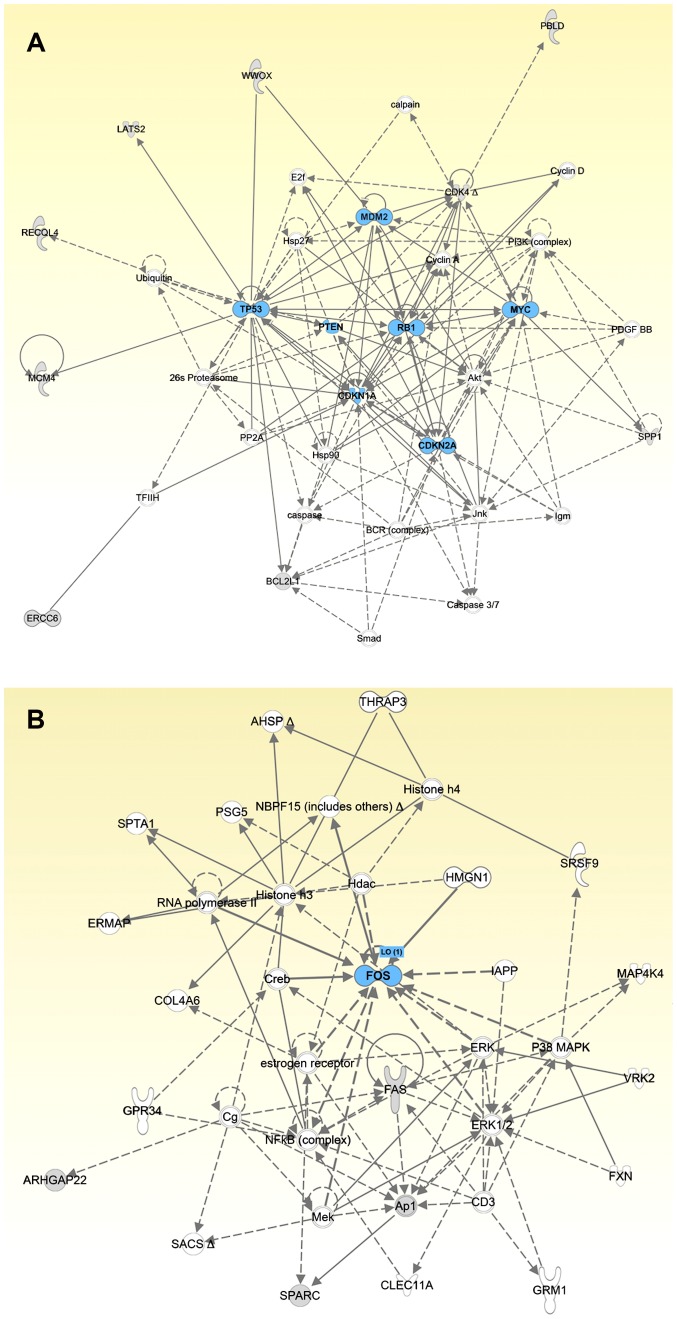
Gene networks generated by Ingenuity Pathway Analysis using the 17 selected genes in the nanoString code set from this study and 31 candidate osteosarcoma driver genes from a previously published data set [32]. Panel A depicts the major over-represented network which has molecular relationships between some of the genes in code set and RB1, TP53, PI3K, PTEN/Akt, MYC, and RECQL4 interactions. Panel B depicts the second ranked network that shows interactions with FOS, FAS, NFkB, and ERK1 signalling pathways.

To investigate the signalling relationship between the 17 selected genes in this study and the 31 candidate osteosarcoma driver genes from a previously published data set [32], standard pathway analysis using IPA software, was performed. Molecular interaction networks explored using IPA tools, allowing a maximum threshold of 35 nodes per network, revealed a total of 15 networks. The top two significant networks are shown in Figures 3A and B. Network 1 included 16 differentially regulated genes (score = 31), with signalling in RB1, TP53, PI3K, PTEN/Akt, MYC, and RECQL4 as the major over-represented gene networks. The interactions between the five-gene signature within signalling network 1 was strong, indicating that there were likely functional interactions of some of the genes with the core cellular regulation of cell cycle control. Network 2 included six genes (score = 8), which are associated with FOS, FAS, NFkB, and ERK1 signalling pathways. These functions are associated with extracellular signalling in the context of bone morphogenesis.

## Discussion

Paediatric osteosarcoma is a rare and complex tumor that has been studied extensively with respect to the genetic alterations that can be present [3]. However, there is still no consistently reliable marker for clinicians to use in prognostication aside from the degree of tumor necrosis in response to chemotherapy, and no targeted therapies exist [33]. Our objective in this study was to characterise a cohort of osteosarcoma tumors using nanoString technology. The nanoString nCounter is a digital expression analysis tool that is increasingly being used to detect and validate molecular signatures distinguishing subgroups of cancers [13]. The efficacy of nanoString molecular bar code analyses relative to traditionally used technologies has been demonstrated by others [34].

Our nanoString expression analyses closely replicated our previous qRT-PCR analyses of an independent cohort of osteosarcoma specimens. Relative to normal human osteoblasts, we detected up-regulation of *CDKN1C*, *FOS*, *MYC*, *RECQL4*, *RUNX2*, *SPARC*, *SPP1*, and *WWOX*, with similar magnitudes of changes for *MYC* and *RUNX2*. The initial IPA analysis of our data, combined with that of Kuijjer et al. [32], demonstrates the potential for disruption of the MAPK/ERK signal transduction pathway via up-regulation of FOS, AP-1, and SPARC (Figure 3B). Relative to normal osteoblasts, changes in the network by aberrant expression of these genes would not only interrupt signal transduction through MAPK/ERK, but affect transcription, DNA stability, and apoptosis regulation.

Up-regulation of *RUNX2*, *CDC5L*, *CDK4*, *RECQL4* and down-regulation of *MDM2* was detected in the tumors that responded poorly to chemotherapy. There was also a statistically significant increase in *RECQL4* expression in tumors relative to normal osteoblasts (Figure S2), a finding that corroborates previous studies from our research group [10]. *RECQL4* overexpression is associated with elevated genome instability in osteosarcoma [30], and genomic instability may, in turn, contribute to chemoresistance and poor prognosis [35]. This gene encodes a DNA helicase important in DNA replication regulation during G(1) and S phases [36]. Germ-line mutations in *RECQL4* are associated with Rothmund-Thomson syndrome, which is linked to the development of several malignancies, including paediatric osteosarcoma [37]. Somatic mutations of *RECQL4* have not been detected in sporadic osteosarcomas, but the present study raises the possibility that genetic amplification may be the method by which *RECQL4* dysfunction presents in sporadic tumors.

In contrast to *RECQL4*, there was minimal change in *CDC5L* or *CDK4* expression between tumors and normal human osteoblasts (Figure 2), as was the case in a previous study by our group [10]. Overexpression of *CDC5L* has been detected in osteosarcoma patient samples and osteosarcoma cell lines alike, and is a probable candidate oncogene within the 6p12-p21 amplicon commonly found in osteosarcomas [9]. CDC5L is an essential component of the spliceosome complex, and elevated CDC5L shortens the G(2)-M cell cycle transition [38]. Furthermore, CDC5L is important in the DNA damage response following exposure to genotoxic agents. It interacts with the checkpoint kinase ATR and is required for activating S-phase checkpoint effectors [39]. The CDK4 protein regulates and promotes cell cycle progression through G1, and elevated levels are common in cancer [40]. *CDK4* amplification and overexpression are commonly found in low-grade [41] and dedifferentiated forms of osteosarcoma [21]. *CDK4* amplification and overexpression tend to be associated with better prognosis in the rarely occurring low-grade osteosarcomas [42] but is associated with poor prognosis in the majority of osteosarcoma cases. [43]. Expression of *MDM2* is known to inhibit *TP53* transcriptional activation [55]. Thus, differential expression of *CDC5L*, *MDM2*, and *CDK4* could contribute to variation in cellular proliferation during osteosarcoma pathogenesis, especially when there is reduced expression of the tumor suppressors RB1 and TP53, or the TP53 target CDKN1A.

These results are also in keeping with a recent report from Kelly et al. [59], who used a similar general experimental approach to identify differentially expressed microRNA associated with chemotherapy response in osteosarcoma. Their study identified a small subset of miRNA and mRNA targets that impacted on some of the same genes and molecular pathways that we identified in this study. For example, there was a strong relationship between some of the gene targets identified in their study and bone morphogenesis proteins such as RUNX2 together with TP53 signaling (Table S4).

Our study also corroborates an expanding body of evidence showing that RUNX2, a transcription factor central to the control of osteoblast differentiation during skeletal development and remodelling, is frequently expressed at high levels in osteosarcoma biopsies [9], [24], [44], [60]. The deregulation of RUNX2 in cancer has been linked to signalling pathways disrupted in tumorigenesis, including RB1 and TP53 [45], [46]. RUNX2 is growth suppressive in normal osteoblasts, but can induce proliferation-specific genes if over-expressed [31]. Loss of TP53 increases protein levels of RUNX2 in osteosarcoma cells by post-transcriptional mechanisms Loss of TP53 prevents post-expression of microRNA miR-34c, which directly targets RUNX2 [47]. However, our study indicates that transcriptional mechanisms may also play an important role.

The *RUNX2* gene is controlled by two promoters. The P1 promoter is activated at late stages of osteoblast differentiation to elevate RUNX2 levels in support of osteoblast maturation [48]. Strikingly, we noted higher levels of expression of the *RUNX2 P2* transcript in tumors which responded poorly to chemotherapy, closely replicating a previous finding from our lab [10]. The P2 promoter contains multiple CpG doublets, and hypomethylation of this promoter may permit expression in mesenchymal stem cells [49]. In addition, *RUNX2* expressed from the P2 promoter regulates hypertrophy and proliferation of both chondrocytes and the closely-related immature osteoblasts [50]–[52]. The human osteosarcoma cell line SAOS-2 has persistently high RUNX2 protein levels, driven by the P2 promoter [53]. Hence, preferential expression of *RUNX2* from the ‘early-activated’ P2 promoter rather than the ‘late-activated’ P1 promoter in osteosarcomas suggests that osteosarcomas may originate from immature mesenchymal progenitor cells.

The samples in this study consisted of tumor resections both from patients who had been treated with chemotherapy and from pre-chemotherapeutic biopsies. Statistically significant gene expression differences between resections and biopsies existed for the expression levels of *CDKN1A*, *MDM2*, *BCL2L1*, *PTEN*, and *WWOX*. *CDKN1A* is activated in response to activation of the ATM/TP53 DNA damage checkpoint that accommodates double-stranded DNA repair and inhibits cell cycle progression by CDK4 [54], MDM2 is an inhibitor of TP53 [55], BCL2L1 is an anti-apoptotic factor [56], PTEN is a tumor suppressor commonly lost in osteosarcomas [23], and WWOX is a tumor suppressor that inhibits RUNX2 activity [24]. Furthermore, all of these genes encode proteins important for cell cycle regulation. The IPA analysis of the data confirms significant relationships between proteins in theTP53-RB1-centred network of protein interactions that also involve PI3K, PTEN/Akt, MYC, and RECQL4 (Figure 3A). All of the aforementioned genes were more highly expressed in the resections. Our results are consistent with experiments in osteosarcoma cell lines that have shown that drug treatment induces growth arrest and increases levels of CDKN1A, MDM2, and BCL2L1 [57], [58].

In conclusion, our results provide preliminary evidence that *RUNX2*, *CDC5L*, *MDM2*, *RECQL4*, and *CDK4* should be further investigated to determine their roles as predictive biomarkers in osteosarcoma, because collectively, their differential expression correlates with poor response to chemotherapy in our cohort.

## Supporting Information

Figure S1 **Cluster map constructed using Cluster 3.0, showing the differential expression of the 16 gene set in osteosarcoma biopsy and resection cases.** Sample numbers 1–36 were analyzed in triplicates whereas, samples 41–116 were analyzed in duplicates. Details of samples are presented in Table S2B. The seven replicates of the three osteosarcoma cell lines and the pooled human osteoblast control used in this comparison cluster to the right as four distinct groupings (C–R). From left to right they are SAOS samples CBAAMLB; MG63 samples GIHQGHP; human osteoblast control samples EDFDEON; and U2OS samples KJLJKSR.
(TIF)

Figure S2 **Expression changes for each of the 32 patient samples were normalized, averaged, and then ratios relative to normal human osteoblast control were calculated.** Statistically significant up-regulated expression in tumors relative to human osteoblasts was detected for *CDKN1C*, *FOS*, *MYC*, *RECQL4*, *RUNX2*, *SPARC*, *SPP1*, and *WWOX.* Down-regulation was observed for *CDKN1A* and *TP53*.
(TIF)

Table S1 **Codeset for expression analysis.**
(XLSX)

Table S2 Table S2A: All nanoString data with normalization applied. Table S2B: Patient nanoString data sets with normalization applied. Patient samples in the first set were analyzed in triplicate, whereas samples in the second and third sets were analyzed in duplicate.
(XLSX)

Table S3 **Gene expression levels differentiated groups of tumors.** The unpaired two-tailed Student’s t-test was applied to derive genes that had significant (p<0.05) differential expression between the group with poor prognosis (n = 25) and the group with good prognosis (n = 15).
(XLSX)

Table S4 **Previously published results show similar pathways of expression deregulation in osteosarcoma.** Genes deregulated in osteosarcoma as identified by Kelly et al. (Additional file 14 Table S5 of reference 59) were functionally related to our experimental genes.
(DOCX)
