# Supplementary figures and images for: Digital Expression Profiling Identifies RUNX2, CDC5L, MDM2, RECQL4, and CDK4 as Potential Predictive Biomarkers for Neo-Adjuvant Chemotherapy Response in Paediatric Osteosarcoma
Source: PLoS One. 2014 May 16;9(5):e95843. doi: 10.1371/journal.pone.0095843 (PMC4023931; doi:10.1371/journal.pone.0095843)

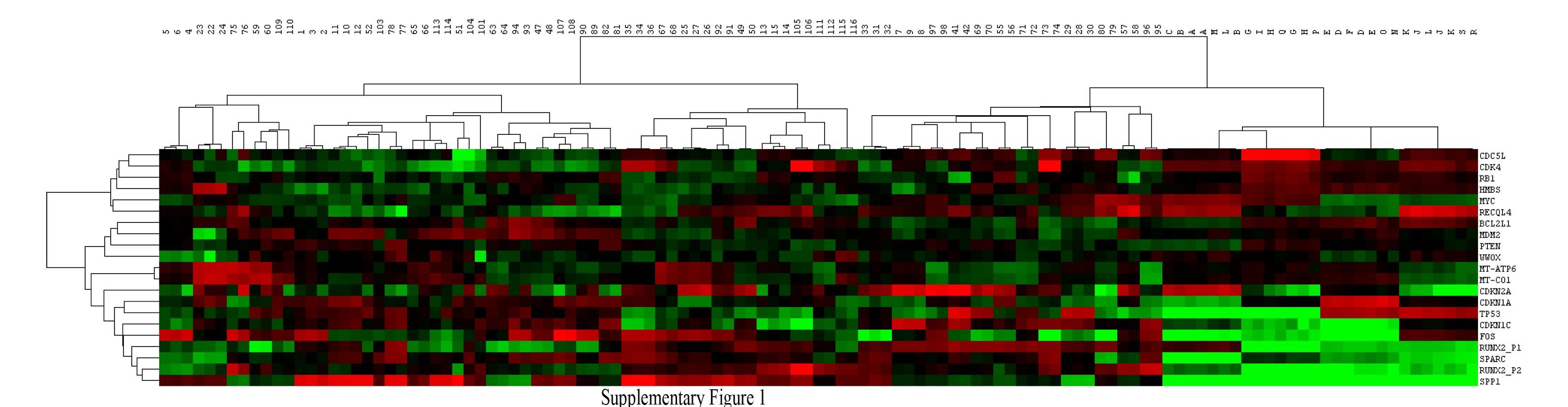

Supplement: Figure S1 — Cluster map constructed using Cluster 3.0, showing the differential expression of the 16 gene set in osteosarcoma biopsy and resection cases. Sample numbers 1–36 were analyzed in triplicates whereas, samples 41–116 were analyzed in duplicates. Details of samples are presented in Table S2B. The seven replicates of the three osteosarcoma cell lines and the pooled human osteoblast control used in this comparison cluster to the right as four distinct groupings (C–R). From left to right they are SAOS samples CBAAMLB; MG63 samples GIHQGHP; human osteoblast control samples EDFDEON; and U2OS samples KJLJKSR. (TIF) [file pone.0095843.s001.tif]

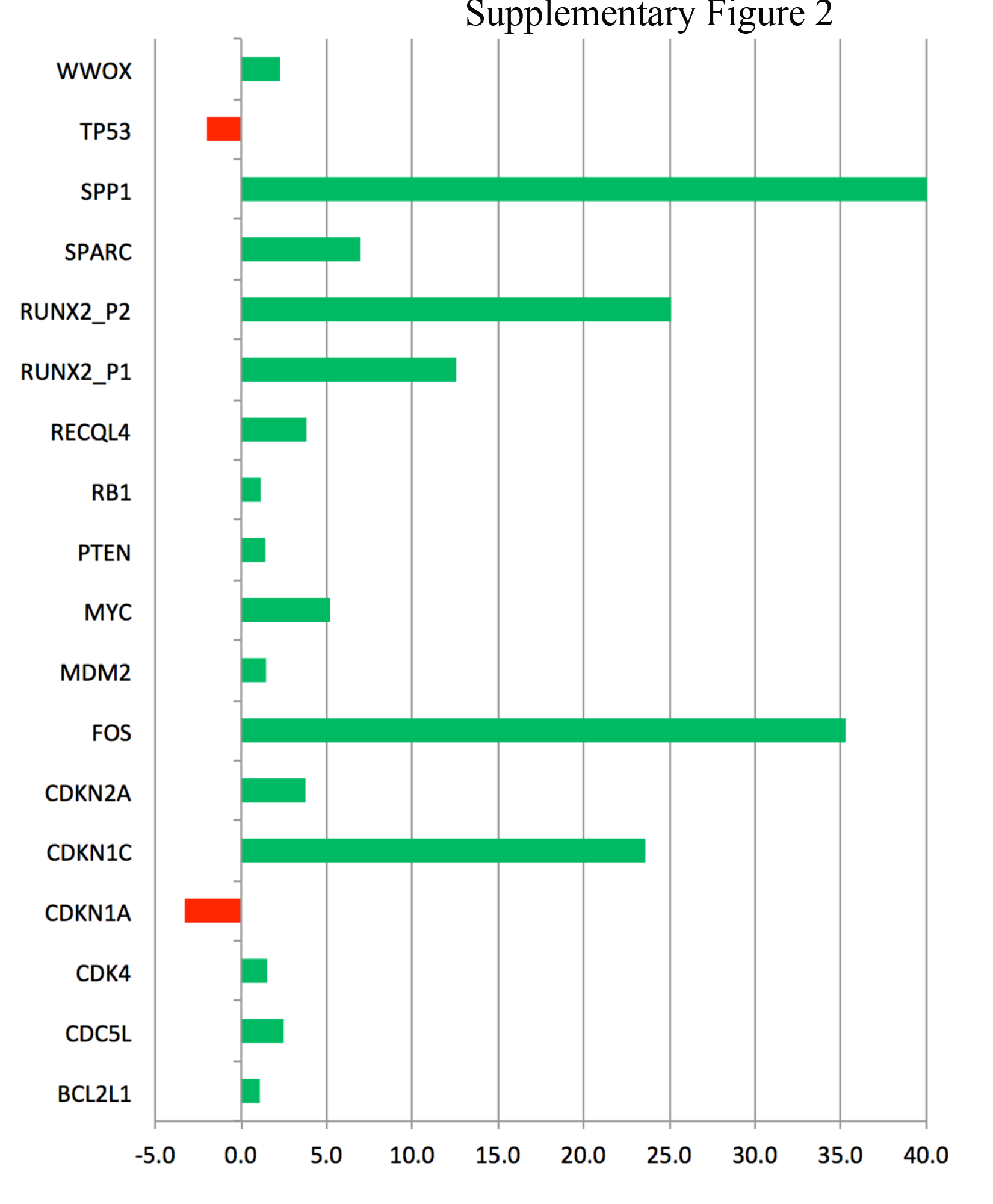

Supplement: Figure S2 — Expression changes for each of the 32 patient samples were normalized, averaged, and then ratios relative to normal human osteoblast control were calculated. Statistically significant up-regulated expression in tumors relative to human osteoblasts was detected for CDKN1C, FOS, MYC, RECQL4, RUNX2, SPARC, SPP1, and WWOX. Down-regulation was observed for CDKN1A and TP53. (TIF) [file pone.0095843.s002.tif]
