# Supplementary material for: Digital Expression Profiling Identifies RUNX2, CDC5L, MDM2, RECQL4, and CDK4 as Potential Predictive Biomarkers for Neo-Adjuvant Chemotherapy Response in Paediatric Osteosarcoma
Source: PLoS One. 2014 May 16;9(5):e95843. doi: 10.1371/journal.pone.0095843 (PMC4023931; doi:10.1371/journal.pone.0095843)
Supplement: Table S4 — Previously published results show similar pathways of expression deregulation in osteosarcoma. Genes deregulated in osteosarcoma as identified by Kelly et al. (Additional file 14 Table S5 of reference 59) were functionally related to our experimental genes. (DOCX) [file pone.0095843.s006.docx]

| **Genes from Kelly, et al.**  **(2013)*** | **Putative function** | **Functional relationship to genes in this study** | **Citations (see next page)** |
| --- | --- | --- | --- |
| *ACAN* | Chondrogenic differentiation | ACAN is associated with RUNX2 expression though bone morphogenetic pathways | 1. Caron, et al. (2013)  2. Kishiya, et al. (2008) |
| *CHAD* | Chondrogenesis, mesenchyme stem cell biology | CHAD and RUNX2 are subjected to coordinated epigenetic regulation | 3. Ezura, et al. (2009)  4. Ehlicke, et al. (2010) |
| *CLDN5 (Claudin-5)* | Endothelial-mesenchyme transition related to calcification | Inverse expression relationship between RUNX 2 and CLDN5 | 5. Cheng, et al. (2013) |
| *JUN* | JUN contributes to API transcription factors | AP1 family of  transcription factors mediate osteoblast differentiation through RUNX2 | 6. Hovhannisyan, et al. (2013)  7. Marie (2008) |
| *BRD4* | Chromatin-associated protein | TP53 partner and potential role in mesenchyme differentiation | 8. den Bakker, et al. (2009)  9. Wu, et al. (2013) |
| *RPL6* | Ribosomal protein | Mediates HDM2-TP53 regulation | 10. Bai, et al. (2013) |

CITATIONS FROM TABLE S4 (Martin, et al.)

1. Caron MM, Emans PJ, Cremers A, Surtel DA, Coolsen MM, van Rhijn LW, Welting TJ. (2013) Hypertrophic differentiation during chondrogenic differentiation of progenitor cells is stimulated by BMP-2 but suppressed by BMP-7. Osteoarthritis Cartilage (4):604-13. doi: 10.1016/j.joca.2013.01.009. Epub 2013 Jan 24. PMID: 23353668

2. Kishiya M, Sawada T, Kanemaru K, Kudo H, Numasawa T, Yokoyama T, Tanaka S, Motomura S, Ueyama K, Harata S, Toh S, Furukawa K. (2008) A functional RNAi screen for Runx2-regulated genes associated with ectopic bone formation in human spinal ligaments. J Pharmacol Sci (3):404-14. Epub 2008 Mar 5. PMID: 18319563

3. Ezura Y, Sekiya I, Koga H, Muneta T, Noda M. (2009) Methylation status of CpG islands in the promoter regions of signature genes during chondrogenesis of human synovium-derived mesenchymal stem cells. Arthritis Rheum 60(5):1416-26. doi: 10.1002/art.24472. PMID: 19404940

4. Ehlicke F, Freimark D, Heil B, Dorresteijn A, Czermak P. (2010) Intervertebral disc regeneration: influence of growth factors on differentiation of human mesenchymal stem cells (hMSC). Int J Artif Organs 33(4):244-52. PMID: 20458694

5. Cheng SL, Shao JS, Behrmann A, Krchma K, Towler DA. (2013) Dkk1 and MSX2-Wnt7b signaling reciprocally regulate the endothelial-mesenchymal transition in aortic endothelial cells. Arterioscler Thromb Vasc Biol 33(7):1679-89. doi: 10.1161/ATVBAHA.113.300647. Epub 2013 May 16. PMID: 23685555

6. Hovhannisyan H, Zhang Y, Hassan MQ, Wu H, Glackin C, Lian JB, Stein JL, Montecino M, Stein GS, van Wijnen AJ. (2013) Genomic occupancy of HLH, AP1 and Runx2 motifs within a nuclease sensitive site of the Runx2 gene. J Cell Physiol 228(2):313-21. doi: 10.1002/jcp.22109. PMID: 2886425

7. Marie PJ. (2008) Transcription factors controlling osteoblastogenesis. Arch Biochem Biophys 473(2):98-105. doi: 10.1016/j.abb.2008.02.030. Epub 2008 Feb 29. Review. PMID: 18331818

8. den Bakker MA, Beverloo BH, van den Heuvel-Eibrink MM, Meeuwis CA, Tan LM, Johnson LA, French CA, van Leenders GJ. (2009) NUT midline carcinoma of the parotid gland with mesenchymal differentiation. Am J Surg Pathol 33(8):1253-8. doi: 10.1097/PAS.0b013e3181abe120. PMID: 19561446

9. Wu SY, Lee AY, Lai HT, Zhang H, Chiang CM. (2013) Phospho switch triggers Brd4 chromatin binding and activator recruitment for gene-specific targeting. Mol Cell 49(5):843-57. doi: 10.1016/j.molcel.2012.12.006. Epub 2013 Jan 11 PMID: 23317504

10. Bai D, Zhang J, Xiao W, Zheng X. (2014) Regulation of the HDM2-p53 pathway by ribosomal protein L6 in response to ribosomal stress. Nucleic Acids Res 42(3):1799-811. doi: 10.1093/nar/gkt971. Epub 2013 Oct 29. PMID: 24174547
